# Accurate and sensitive interactome profiling using a quantitative protein-fragment complementation assay

**DOI:** 10.1016/j.crmeth.2024.100880

**Published:** 2024-10-21

**Authors:** Natalia Lazarewicz, Gaëlle Le Dez, Romina Cerjani, Lunelys Runeshaw, Matthias Meurer, Michael Knop, Robert Wysocki, Gwenaël Rabut

**Affiliations:** 1 University Rennes, CNRS, INSERM, Institut de Génétique et Développement de Rennes (IGDR), UMR6290, U1305, Rennes, France; 2 Department of Genetics and Cell Physiology, Faculty of Biological Sciences, University of Wroclaw, Wroclaw, Poland; 3 Zentrum für Molekulare Biologie der Universität Heidelberg (ZMBH), DKFZ-ZMBH Alliance, Heidelberg, Germany

**Keywords:** protein-protein interaction, interactome, NanoBiT, Cdc53, Irc20, Met30, Nam7, Upf1, *Saccharomyces cerevisiae*, budding yeast

## Abstract

An accurate description of protein-protein interaction (PPI) networks is key to understanding the molecular mechanisms underlying cellular systems. Here, we constructed genome-wide libraries of yeast strains to systematically probe protein-protein interactions using NanoLuc Binary Technology (NanoBiT), a quantitative protein-fragment complementation assay (PCA) based on the NanoLuc luciferase. By investigating an array of well-documented PPIs as well as the interactome of four proteins with varying levels of characterization—including the well-studied nonsense-mediated mRNA decay (NMD) regulator Upf1 and the SCF complex subunits Cdc53 and Met30—we demonstrate that ratiometric NanoBiT measurements enable highly precise and sensitive mapping of PPIs. This work provides a foundation for employing NanoBiT in the assembly of more comprehensive and accurate protein interaction maps as well as in their functional investigation.

## Highlights

• Establishes ratiometric NanoBiT as an accurate method to explore interactomes
• Provides genome-wide yeast strain libraries to conduct systematic NanoBiT assays
• Showcases NanoBiT’s performance by analyzing Upf1, Cdc53, and Met30 interactomes
• Illustrates NanoBiT’s use for functional study of protein-protein interaction networks

## Motivation

Characterization of protein-protein interaction networks is essential to understanding cellular functions and diseases. However, current interactomic methods capture only a fraction of the entire cellular interactome. For example, the yeast two-hybrid method is limited to detecting direct interactions, while affinity purification-mass spectrometry often miss transient interactions crucial to understanding dynamic cellular processes. Here, we evaluate the performance of NanoBiT, a protein-fragment complementation assay capable of detecting both direct and indirect, stable and transient protein-protein interactions in live cells, as a complementary method to investigate interactomes.

## Graphical abstract

**Figure:**
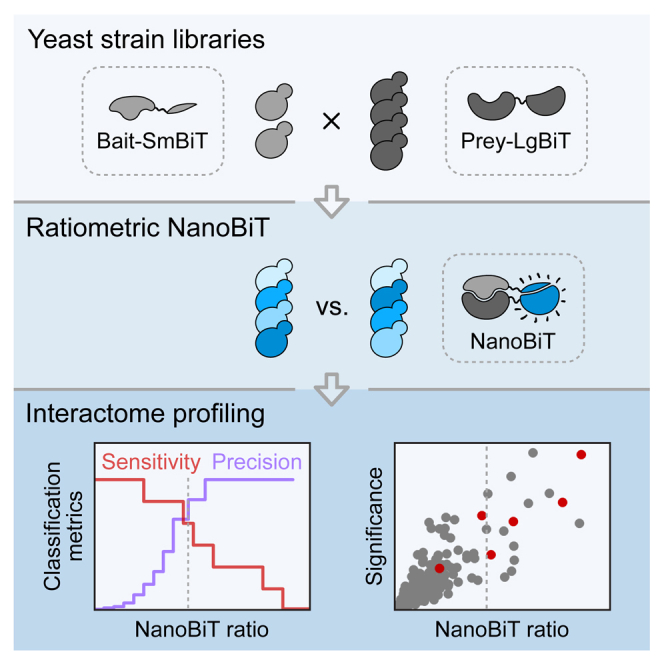

## Teaser

Lazarewicz et al. develop yeast strain libraries to monitor protein-protein interactions in live cells using NanoBiT, a quantitative luciferase-based assay. They establish that ratiometric NanoBiT measurements enable accurate interactome investigation with a sensitivity equivalent to or better than that of other proteomic approaches while providing complementary results.

## Introduction

Protein-protein interactions (PPIs) underlie most cellular processes and are frequently perturbed by disease-associated mutations.^1^^,^^2^ Over the past two decades, significant efforts have been devoted to assembling proteome-wide PPI maps using various experimental strategies.^3^^,^^4^^,^^5^^,^^6^^,^^7^^,^^8^^,^^9^^,^^10^^,^^11^ However, current assays used in interactomic studies only detect subsets of the entire cellular interactome.^12^^,^^13^^,^^14^ Typically, when performed under conditions that limit the detection of negative control proteins, state-of-the-art binary PPI assays detect up to a third of well-described benchmark interactions.^10^^,^^12^ A combination of multiple assays is therefore necessary to increase overall PPI detection and obtain a proper interactome coverage.

**Figure 1:**
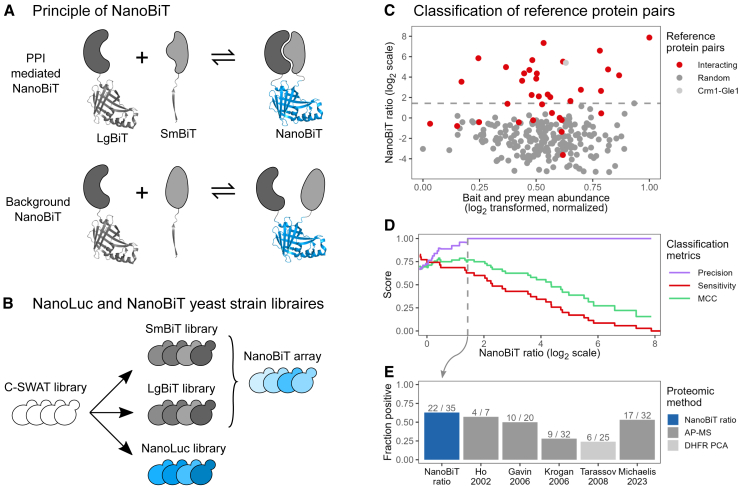
Benchmarking NanoBiT for detecting the interaction of endogenously tagged protein pairs (A) Direct and indirect interactions between proteins fused to LgBiT and SmBiT fragments facilitate the reconstitution of the NanoBiT enzyme. Self-association of LgBiT and SmBiT can also lead to background NanoBiT reconstitution, depending on the abundance and subcellular localization of the LgBiT- and SmBiT-tagged proteins. PPI, protein-protein interaction. (B) The SWAT strategy^23^ was used to construct libraries of SmBiT- and LgBiT-tagged strains of opposite mating types along with a library of NanoLuc-tagged strains. (C) Scatterplot showing the NanoBiT ratio (y axis) and the mean of NanoLuc intensities (x axis) exhibited by reference protein pairs. Interacting pairs are shown in red, random pairs in gray, and the Crm1-Gle1 pair in light gray. Data are from four experimental replicates. The dashed line indicates the highest NanoBiT ratio exhibited by the random pairs. (D) Step chart representing the precision, sensitivity, and Matthews correlation coefficient (MCC) for the classification of reference pairs across varying NanoBiT ratios. The dashed line indicates the highest NanoBiT ratio exhibited by the random pairs. (E) Fraction of reference interacting pairs detected by ratiometric NanoBiT and in landmark large-scale interactome studies.^5^^,^^6^^,^^24^^,^^25^^,^^26^

Protein-fragment complementation assays (PCAs) are a set of related methods widely used to probe the interaction of protein pairs in their native cellular context.^15^^,^^16^ They rely on rationally designed complementary fragments of a reporter protein, which are genetically fused to bait and prey proteins. Interaction between the bait and the prey enhances the spatial proximity of the reporter fragments and facilitates reporter reconstitution. Diverse PCA reporters have been established, including the dihydrofolate reductase (DHFR), fluorescent proteins, and luciferases.^16^^,^^17^^,^^18^^,^^19^^,^^20^^,^^21^ Among them, the NanoLuc Binary Technology (NanoBiT) is particularly appealing for interactome studies. It is derived from NanoLuc, a 19 kDa luciferase that, in presence of its optimized substrate, furimazine, produces a sustained luminescence about 100-fold more intense than that of other luciferases.^22^ The NanoBiT fragments, termed Large BiT (LgBiT) and Small BiT (SmBiT), were specifically engineered for low affinity (K_D_ = 190 μM),^19^ thus reducing their direct self-association (Figure 1A). These properties (bright luminescence and low affinity of the LgBiT and SmBiT fragments) promise a high sensitivity and reduced background of NanoBiT compared to other PCA reporters.

Here, we evaluate the performance of NanoBiT for interactomic studies using the model organism *Saccharomyces cerevisiae*. We constructed genome-wide libraries of yeast strains and used them to probe a reference set of 35 interacting and 206 random PPIs, as well as the interactome of four proteins with different levels of characterization. Notably, ratiometric NanoBiT measurements detected well-described PPIs with high precision and sensitivity levels matching or exceeding those obtained in previous large-scale studies. Overall, our results demonstrate that NanoBiT enables systematic and functional interactome investigations with remarkable accuracy.

## Results

### Benchmarking NanoBiT for detecting the interaction of endogenously tagged protein pairs

In order to empower the use of NanoBiT in interactome studies, we first constructed libraries of yeast strains using the SWAp-Tag (SWAT) method (Figure 1B).^23^ This method employs a library of 5,661 yeast strains containing a SWAT acceptor module integrated before the stop codon of individual open reading frames (ORFs). Using successive selections on appropriate media, the acceptor module can be efficiently exchanged with a donor module provided by a replicative plasmid. We previously used this method to construct a library of *MAT*alpha strains harboring LgBiT-tagged ORFs flanked by a hygromycin B resistance cassette.^27^ We now adapted the selection procedure to construct a complementary library of *MAT*a strains with SmBiT-tagged ORFs marked with a nourseothricin resistance cassette (see details in the STAR Methods section and Table S1). The strains from these two libraries can then be crossed to derive diploid or haploid strains expressing any combination of LgBiT- and SmBiT-tagged proteins through selection on appropriate media. Furthermore, we similarly constructed an analogous library of NanoLuc-tagged strains, enabling us to quantify the abundance of the tagged proteins in cells with an identical genetic background.

We employed these libraries to construct NanoBiT strains of reference interacting and non-interacting protein pairs. For the interacting proteins, we randomly picked 35 homo- or heteromeric pairs previously described by both X-ray crystallography and yeast two-hybrid assay, most of which had undergone extensive validation (Table S2). The proteins composing these pairs were shuffled to generate 206 random, likely non-interacting pairs. We crossed the corresponding strains from the LgBiT and SmBiT libraries to produce an array of diploid (for homomeric pairs) or haploid (for heteromeric pairs) NanoBiT strains. The luminescence of these strains was then measured along with that of NanoLuc strains corresponding to each of the tagged proteins from the NanoBiT strains. In both cases, the measurements were highly reproducible across multiple replicates (Pearson correlation coefficient *R* ≥ 0.93, Figure S1A), demonstrating the reliability of our experimental setup.

Overall, the luminescence intensities produced by most strains from the interacting set were higher than those of the strains from the random set (Figure S1B). One notable exception was the Crm1-Gle1 NanoBiT strain, which exhibited high luminescence, although Crm1 and Gle1 had not been reported to interact. However, considering that Gle1 localizes to nuclear pore complexes^28^ and Crm1 mediates the nuclear export of numerous proteins and ribonucleoproteins (RNPs),^29^ it is highly probable that the NanoBiT signal produced in this strain is actually specific. Excluding this particular case, 14% of the strains from the random set produced luminescence intensities above our luminometer’s detection limit (Figure S1B). These random pairs that produced detectable luminescence mainly consisted of abundant proteins. This observation suggests that in some strains from the random set, NanoBiT signals actually arise through the self-association of LgBiT and SmBiT (Figure 1A).

We then set out to establish an optimal method for the classification of NanoBiT results. A basic approach consists of directly using NanoBiT luminescence intensities as a classifier and choosing a threshold that maximizes the detection of interacting pairs while minimizing the detection of random pairs.^30^ While this method exhibited good sensitivity (the fraction of interacting pairs scoring positive) and precision (the fraction interacting pairs among all of those that score positives) (Figure S1C), we reasoned that it might not be the most effective approach. Unrelated protein pairs with markedly different abundance or subcellular localization are likely to exhibit varying levels of LgBiT/SmBiT self-association. As a result, a classifier directly using luminescence intensities might overlook pairs of truly interacting but weakly expressed proteins, potentially yielding lower luminescence signals than more abundant non-interacting pairs. To address this concern, we explored an alternative classifier based on the ratio of luminescence exhibited by related pairs sharing one partner. For each of the investigated pairs, we computed the ratio of its luminescence to the maximum luminescence observed for random pairs (excluding Crm1-Gle1) containing either the bait or the prey from the original pair. This ratiometric classifier appeared less skewed by protein abundance than NanoBiT signals (Figures 1C and S1B) and improved the classification results (Figures 1D and S1C). At 100% precision, ensuring that none of the random pairs (except Crm1-Gle1) scored positive, it enabled the identification of 22 of the interacting pairs from the reference set, corresponding to a sensitivity of 62%. This sensitivity is at least as good as that of several previous landmark interactome studies based on mass spectrometry approaches^5^^,^^24^^,^^25^^,^^26^ or the DHFR PCA reporter^6^ (Figures 1E and S1D). Altogether, these results indicate that NanoBiT experiments enable capturing the interaction of a diverse set of endogenously tagged proteins with high accuracy.

### Benchmarking NanoBiT for systematic interactome profiling

We then aimed to evaluate the performance of NanoBiT for systematically exploring the interactome of proteins of interest. We selected four proteins with varying levels of characterization regarding their molecular functions and interaction partners: Upf1/Nam7, a central component of the nonsense-mediated mRNA decay (NMD) machinery^31^^,^^32^; Cdc53, a cullin family protein that serves as a scaffold for the assembly of SCF ubiquitin ligase complexes^33^^,^^34^; Met30, an F-box domain substrate adaptor of SCF complexes, which controls the transcriptional activation of *MET* genes^35^^,^^36^; and Irc20, a putative ubiquitin ligase and DNA translocase whose partners remain elusive^37^^,^^38^. The corresponding SmBiT-tagged strains were crossed with the entire LgBiT library, and their haploid progeny was isolated on selective media (Table S1).

High-throughput luminescence measurements of the obtained NanoBiT strains were performed by transferring SBS-format arrayed colonies into 384-well microtiter plates filled with culture medium containing furimazine. The luminescence of the NanoLuc library was measured using the same procedure. The luminescence levels of the NanoLuc strains were well above the luminometer detection limit and highly correlated between experimental replicates (*R* ≥ 0.98, Figure S2A). In contrast, most NanoBiT strains exhibited low luminescence intensities, often indistinguishable from the detection limit, indicative of a low rate of self-association between LgBiT and SmBiT. Overall, the Upf1 NanoBiT strains produced the highest signals and the Irc20 strains the lowest (Figures S2A–S2C). Importantly, we observed that the LgBiT-tagged preys exhibiting well-detectable NanoBiT signals were primarily among the most abundant proteins (Figures S2B and S2C). Furthermore, the NanoBiT signals generated by the four SmBiT-tagged baits were correlated with each other (Figure S2B), indicating that identical preys produced NanoBiT signals regardless of the bait. This correlation was highest between Upf1 and Cdc53 NanoBiT data (*R* = 0.71), although Cdc53 and Upf1 are not described to share major interaction partners except the ubiquitin-conjugating enzyme Cdc34^39^^,^^40^^,^^41^ (Table S3). Altogether, these results confirm our previous observation with the random reference pairs. Despite their minimal affinity, the self-association of LgBiT and SmBiT generates detectable background luminescence across multiple protein pair combinations, especially when the tagged proteins are abundant. This background luminescence needs to be controlled and taken into account for an accurate interpretation of NanoBiT data.

To determine the best classification method for analyzing these systematic NanoBiT experiments, we focused on the data obtained with Upf1 and Cdc53, which are the two baits displaying the largest number of well-characterized interactors (Table S3). For sensitivity estimation, we selected high-confidence literature-curated interactors of Cdc53 and Upf1 supported by three or more experimental pieces of evidence. Conversely, all proteins not previously reported to interact with either Upf1 or Cdc53 and lacking Gene Ontology (GO) annotations related to the known functions of Upf1 or Cdc53 were considered as likely non-interactors. Contrary to our previous results with the reference set, the direct use of NanoBiT luminescence intensities as a classifier did not achieve a good level of precision (Figures S3A and S3B), as multiple abundant likely non-interactors of Upf1 or Cdc53 exhibited luminescence intensities comparable to or higher than high-confidence interactors (Figure S2C). In contrast, employing ratiometric classifiers yielded effective classifications. We first calculated NanoBiT ratios for each bait-prey combination, using as a control the signal observed for the same prey with a different bait. The best classification of Upf1 interactors was achieved when employing Cdc53 data as controls (Figure S3A). Conversely, the best classification of Cdc53 interactors was obtained by using Upf1 data for ratio calculations (Figure S3B). This result is consistent with the observation that Upf1 and Cdc53 NanoBiT signals displayed the strongest correlation, suggesting that several preys generate background luminescence with both baits (Figure S2B). We then tested whether including multiple baits as controls, instead of a single one, would enhance the classification results. Met30 was excluded as a control for classifying Cdc53 data since both proteins interact in SCF^Met30^ complexes. We found that while it had little effect on the results obtained for Upf1, it improved the precision of Cdc53 interactor identification (Figures S3A and S3B). We therefore opted to use multiple controls for further analysis of the NanoBiT datasets.

**Figure 2:**
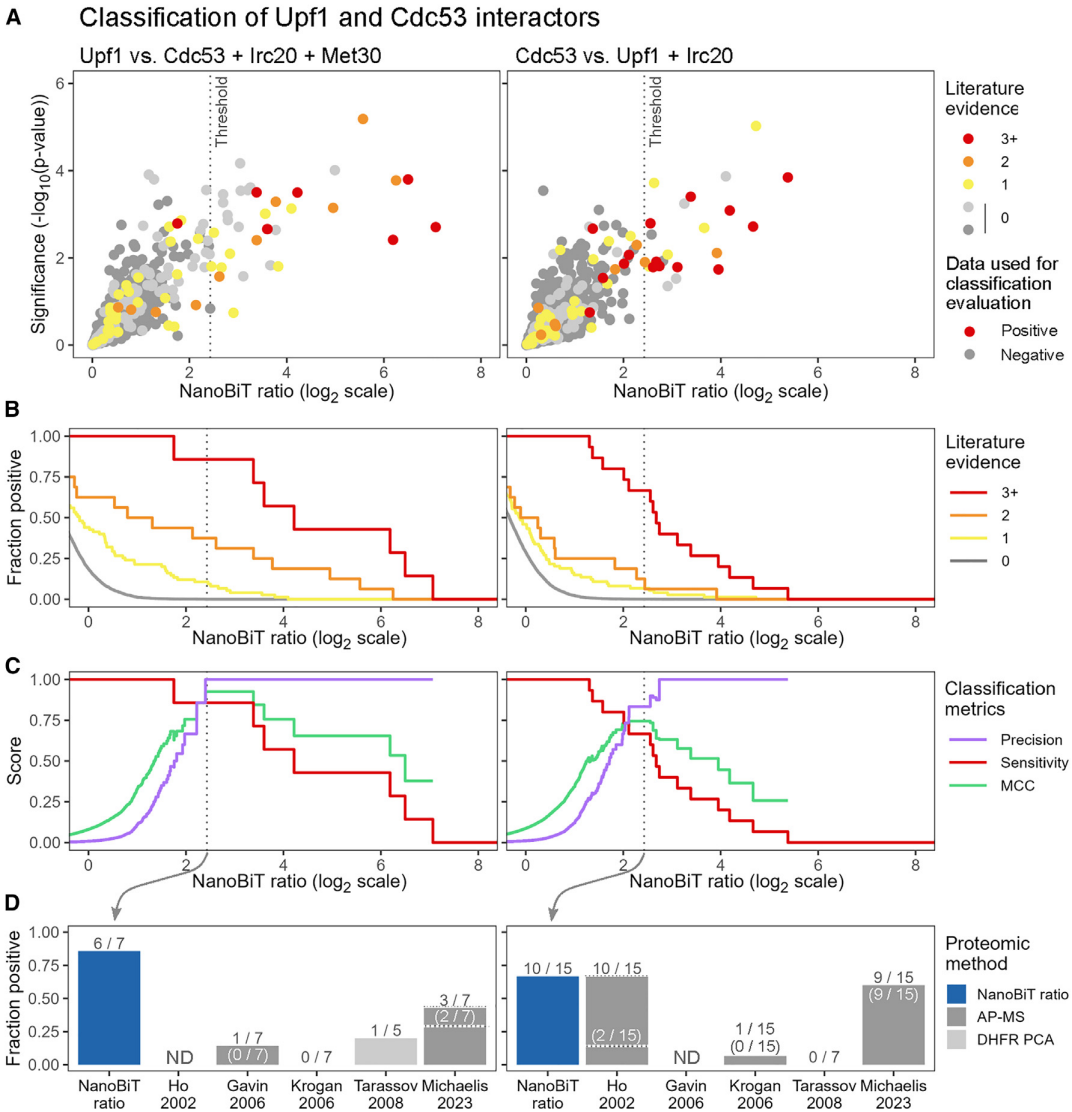
Benchmarking NanoBiT for systematic interactome profiling (A) Scatterplots representing the ratio (x axis) and the significance of the difference (y axis) of NanoBiT signals exhibited by Upf1 (left) and Cdc53 (right) strains versus the indicated control strains. Data are from three experimental replicates. Colors indicate the amount of experimental evidence for each protein pair, as well as which data points were used for classification metrics’ calculation. The dotted line indicates the selected classification threshold. (B) Step chart representing the fraction of Upf1 and Cdc53 literature-curated interactors classified as positive across varying NanoBiT ratios. (C) Step chart representing the precision, sensitivity, and Matthews correlation coefficient (MCC) for the classification of high-confidence Upf1 and Cdc53 interactors (i.e., supported by three or more experimental pieces of evidence) across varying NanoBiT ratios. (D) Fraction of the high-confidence Upf1 and Cdc53 interactors detected by ratiometric NanoBiT and in large-scale interactome studies.^5^^,^^6^^,^^24^^,^^25^^,^^26^ For these studies, the number of interactors detected using Upf1 and Cdc53 as bait (and not as prey) is indicated in brackets.

To identify a classification threshold that maximizes the detection of true positive interactors while minimizing false positives, we computed the Matthews correlation coefficient (MCC) for both Upf1 and Cdc53 classifications. The MCC provides a balanced assessment of binary classifications by taking into account all possible outcomes (true positives, true negatives, false positives, and false negatives).^42^ We selected a NanoBiT ratio threshold corresponding to the maximum MCC of both Upf1 and Cdc53 classifications (Figure S3C). Importantly, almost all bait-prey pairs above this threshold exhibited NanoBiT signals significantly different from those of the control pairs (*p* < 0.05, Figure 2A). For high-confidence interactors of Upf1 and Cdc53 supported by at least 3 pieces of experimental evidence, this threshold achieved sensitivities and precisions exceeding 65% and 80%, respectively (Figure 2C). The detection rate of previously described interactors supported by less evidence was poorer (Figure 2B). This result, consistent with previous observations,^7^ suggests that some of these less described interactors may actually be false positives. Importantly, the sensitivity obtained for high-confidence interactors was as good as or better than that in previous interactome studies^5^^,^^6^^,^^24^^,^^25^^,^^26^ (Figure 2D; Table S2). Overall, our results indicate that systematic ratiometric NanoBiT experiments can effectively capture protein interactomes.

### Description of Cdc53, Met30, and Upf1 interactomes revealed by NanoBiT

**Figure 3:**
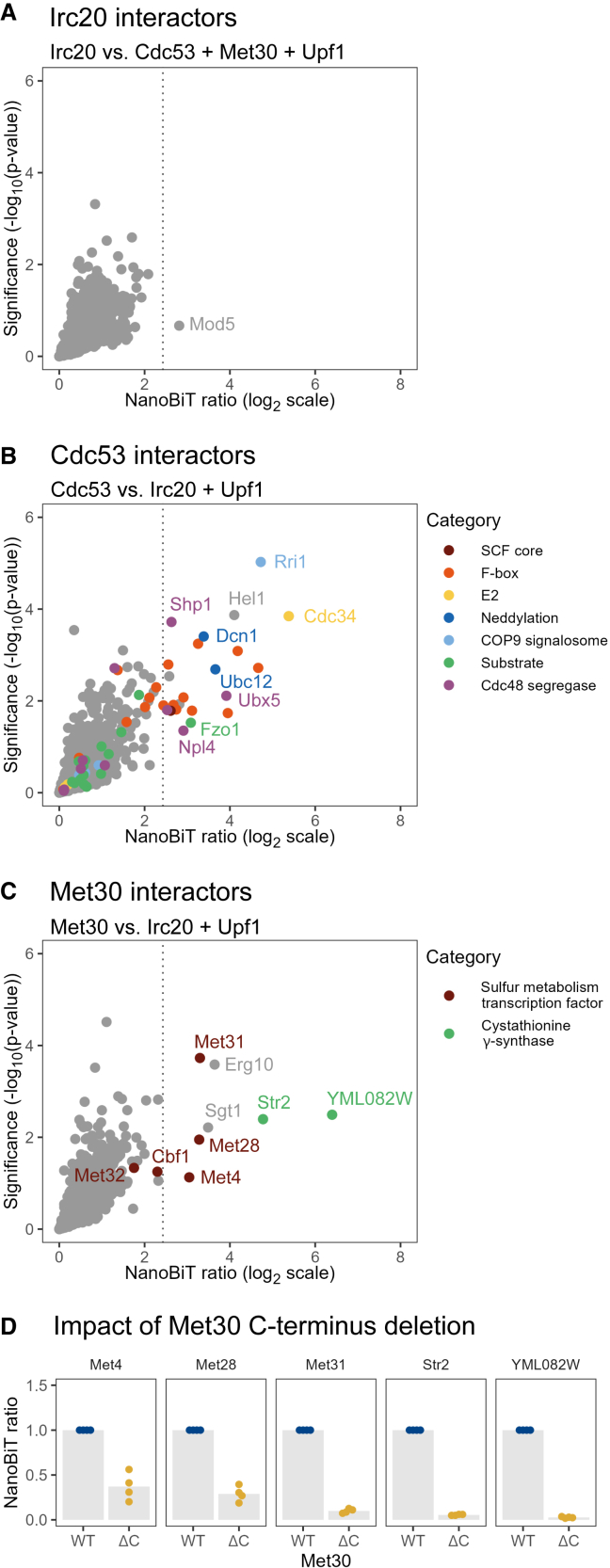
Irc20, Cdc53, and Met30 interactors detected by NanoBiT (A–C) Scatterplots representing the ratio (x axis) and the significance of the difference (y axis) of NanoBiT signals exhibited by the indicated strains. Data are from three experimental replicates. The dotted line indicates the selected classification threshold. Different colors represent categories of Cdc53 and Met30 interactors. (D) Relative NanoBiT signals exhibited by strains expressing either wild-type (WT) or C-terminally truncated (ΔC, amino acids [aa] 1–227) SmBiT-tagged Met30 and the indicated LgBiT-tagged proteins. NanoBiT ratios were computed using WT cells as controls. Data are from four experimental replicates.

We proceeded to investigate the molecular nature of the interactors of each bait. At the optimal classification threshold, we identified 33, 23, and 7 high-confidence interactors of Upf1, Cdc53, and Met30, respectively, including both known and undescribed interaction partners (Table S3). In contrast, a single prey, the tRNA modifier Mod5, scored positive for Irc20 interaction but with low significance (Figure 3A; Table S3).

GO enrichment analysis revealed that Cdc53 interactors were significantly enriched in proteins annotated with terms related to the ubiquitin proteasome system (UPS), such as “proteolysis” or “SCF ubiquitin ligase complex” (Figure S4; Table S4). Indeed, manual examination indicated that 21 of the 23 identified interactors are functionally linked to SCF ubiquitin ligases and the UPS (Figure 3B). These include SCF subunits (the RING protein Hrt1 and 10 F-box substrate adaptors) and proteins that transiently interact with SCF complexes, such as the ubiquitin-conjugating enzyme (E2) Cdc34, components of the neddylation/deneddylation machinery (Ubc12, Dcn1, Rri1), and an SCF ubiquitylation substrate (Fzo1) (Figure 3B; Table S3). Interestingly, we also observed interactions between Cdc53 and 4 substrate-recruiting cofactors of the Cdc48 seggregase (Ubx5, Shp1, Ufd1, and Npl4) as well as Hel1, a RING-in-between-RING (RBR) ubiquitin ligase of the Ariadne family.

Concerning Met30, 5 of the 7 identified interactors were annotated as involved in “sulfur amino acid metabolism” (Figure S4; Table S4). Among them are Met4, the master transcriptional regulator of sulfur metabolism, and two of its DNA-binding cofactors, Met28 and Met31 (Figure 3C; Table S3). These interactors were expected, as Met30 is well known to direct Met4 ubiquitylation, thereby controlling the activity of Met4-containing transcription factor complexes.^35^^,^^36^^,^^43^ The two other interactors annotated as involved in sulfur amino acid metabolism are Str2, an enzyme of the reverse transsulfuration pathway,^44^ and its paralog, YML082W. Although both proteins have been reported to interact with Met30 in previous interactome studies,^26^^,^^45^ they have not yet been functionally linked to Met30. To validate these interactions, we constructed yeast strains expressing a truncated version of SmBiT-tagged Met30 lacking the C-terminal domain required for Met4 interaction. This truncation largely reduced the NanoBiT signal produced by Met30 with Met4, Met28, Met31, Str2, and YML082W (Figure 3D), suggesting that Str2 and YML082W are genuine interactors of Met30. The two remaining proteins that scored positive, Sgt1 and Erg10 (Figure 3C), have not, to our knowledge, been linked to Met30. Sgt1 is a conserved and essential co-chaperone that interacts with certain SCF complexes via the Skp1 linker protein.^46^ Erg10 is a central enzyme in acetyl-coenzyme A (CoA) and sterol metabolism,^47^ suggesting a possible connection between sulfur and acetyl-CoA metabolism.

**Figure 4:**
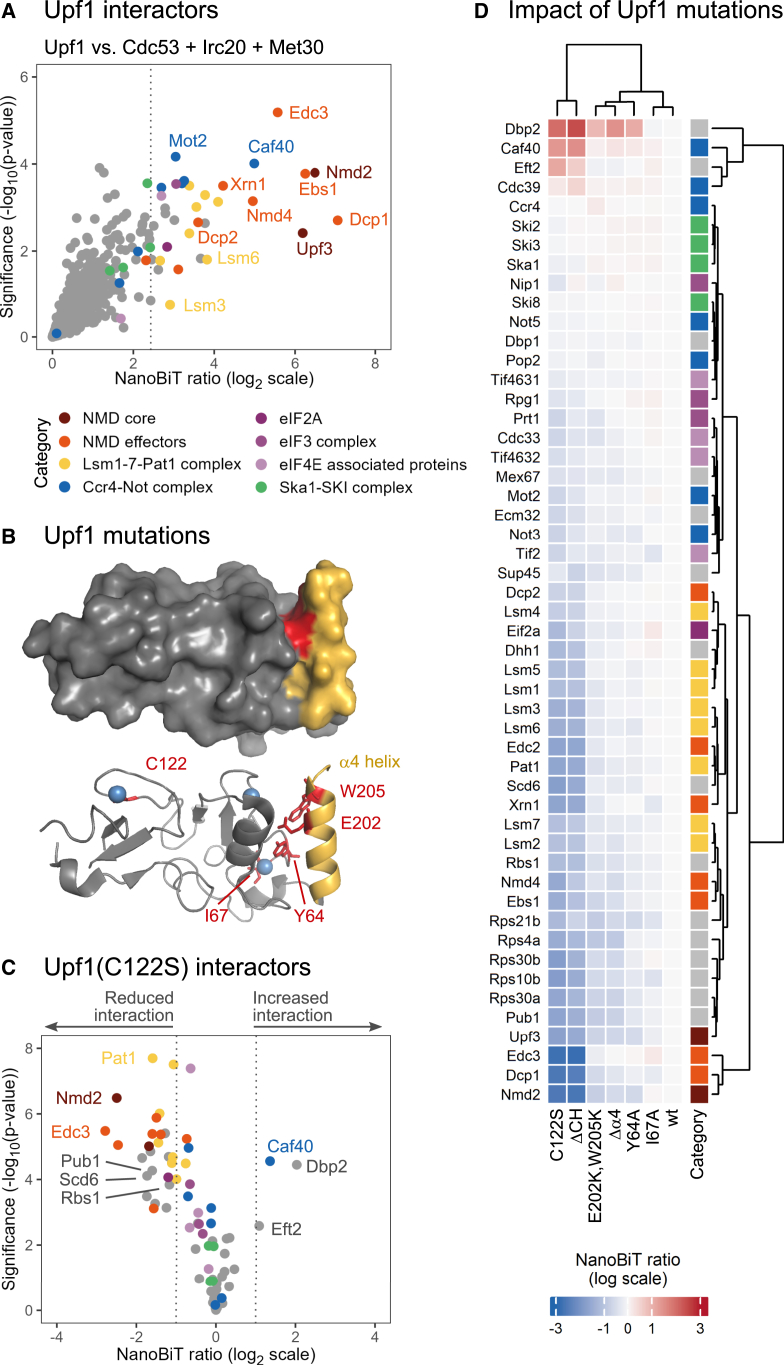
Influence of the CH domain of Upf1 on its interactome (A) Scatterplot representing the ratio (x axis) and the significance of the difference (y axis) of NanoBiT signals exhibited by Upf1 strains versus the indicated control strains. Data are from three experimental replicates. The dotted line indicates the selected classification threshold. Different colors represent categories of Upf1 interactors. (B) Representations of the Upf1 CH domain structure (PDB: 2XZL). Mutated residues are shown in red, the α4 helix of the CH domain in yellow, and zinc atoms in blue. (C) Volcano plot representing changes in NanoBiT signals induced by the C122S mutation for 51 prey proteins. Data are from four experimental replicates. The dashed lines indicate a 2-fold increase or decrease in NanoBiT signals. Upf1 interactors are color coded as in (A). (D) Heatmap representing the changes in NanoBiT signals induced by the indicated Upf1 mutations for 51 prey proteins. Data are from four experimental replicates. Upf1 interactors are color coded as in (A).

In accordance with the well-described role of Upf1 as the primary regulator of NMD, the interactors of Upf1 that we identified were significantly enriched in proteins known to function in mRNA decay or translation regulation and that localize to P-bodies (Figure S4; Table S4). Notably, interaction signals were detected with all but one of the proteins composing two previously described Upf1-containing complexes, namely the NMD core complex^32^ (we observed interactions with Nmd2/Upf2 and Upf3) and the Upf1-decapping complex^48^ (we observed interactions with Dcp1, Dcp2, Edc3, Nmd4, and Ebs1) (Figure 4A; Table S3). We also detected interactions between Upf1 and multiple proteins involved in NMD execution, such as the decapping activator Edc2, the 5′-to-3′ exoribonuclease Xrn1, five subunits of the Ccr4-Not deadenylation complex,^49^ and all subunits of the Lsm1-7-Pat1 complex, which is important for coupling mRNA deadenylation, decapping, and degradation.^50^^,^^51^ The other Upf1 interactors that we detected were proteins of the small ribosomal subunit, translation initiation factors, and further proteins involved in translation or mRNA decay regulation (Scd6, Pub1, Psp2, Puf3). Thus, Upf1 interactors identified using NanoBiT appear to reflect diverse stages of NMD execution, from the assembly of the NMD core complex to mRNA degradation.

### Influence of the CH domain of Upf1 on its interactome

Upf1 harbors at its N terminus a cysteine- and histidine-rich (CH) domain, which is essential for NMD and mediates multiple PPIs. Notably, this domain interacts directly with Nmd2, assembling the NMD core complex together with Upf3.^32^ It was also shown to self-associate^52^ and directly interact with other proteins such as the decapping enzyme subunit Dcp2.^53^^,^^54^^,^^55^ Some of these interactions are mutually exclusive, indicating that the Upf1 CH domain coordinates the assembly of several complexes involved in NMD execution.^48^ Moreover, the CH domain contains two modules related to the RING domain of ubiquitin ligases^56^ and was suggested to catalyze protein ubiquitylation.^41^ Yet, the possible role of this ubiquitin ligase activity in NMD remains unresolved.

In order to further analyze PPIs mediated by the Upf1 CH domain, we constructed yeast strains expressing different mutants of this domain. Two mutations were designed to disrupt the structure of the CH domain: a deletion of residues 62–152 (ΔCH) and a mutation of a structural cysteine residue that coordinates with a zinc atom (C122S) (Figure 4B). Additional mutations were introduced into the putative E2 interaction surface of the RING-like module 1 and the C-terminal helix α4, which partially occludes this surface (Y64A, I67A, E202K-W205K, Δα4)^56^ (Figure 4B). The mutant strains were crossed with strains expressing 51 LgBiT-tagged preys, comprising proteins classified as Upf1 interactors in the systematic NanoBiT screen (Table S4) as well as a selection of other putative Upf1 interactors. The ΔCH and C122S mutations had the strongest impact on NanoBiT signals (Figures 4C and 4D). The I67A mutation showed no significant effect, while the other mutations had a modest impact. Notably, the Y64A mutation, intended to impair the putative E2 interaction of the RING-like module 1, did not alter the Upf1 interactome in a strong or distinctive way. Remarkably, hierarchical clustering of NanoBiT ratios grouped prey proteins according to their described molecular activity (Figure 4D).

## Discussion

Scoring methods play a determinant role in the interpretation of experimental data from protein interaction assays. For affinity purification-mass spectrometry (AP-MS) experiments, sophisticated statistical approaches have been developed to analyze spectral counts and differentiate specific interactors of a given bait from contaminants.^57^^,^^58^ In binary assays where PPIs are probed in pairs, such as PCAs or yeast two-hybrid assays, high-confidence interactions are typically identified by setting a signal intensity threshold above which well-documented PPIs can be reliably detected.^30^ For NanoBiT assays performed with endogenously tagged proteins, we observed that while this approach can yield satisfactory results when probing a limited number of PPIs, such as our reference set of interacting and likely non-interacting pairs (Figures S1B and S1C), it fails to produce an efficient classification when systematically profiling the interactome of bait proteins like Upf1 or Cdc53 (Figures S3A and S3B). This is because certain abundant preys generate higher background luminescence than less-abundant genuine partners of the baits (Figure S2C), due to the self-association of LgBiT and SmBiT. A previous large-scale study performed with the DHFR PCA reporter similarly reported that 427 proteins, mainly highly expressed, displayed spurious PCA signals.^6^ To obtain satisfactory classifications, the authors excluded these proteins from their analysis. Here, we demonstrate that efficient classifications can be achieved using ratiometric NanoBiT measurements, where the luminescence signals obtained with a given bait are normalized against those exhibited by one or multiple control baits. Importantly, the choice of the control(s) influences the classification performance (Figures S3A and S3B). Ideal controls should share the same subcellular localization and have a similar abundance as the bait of interest, while displaying disitinct interactomes. In practice, our results indicate that effective classifications can be obtained when the NanoBiT signals exhibited by the investigated bait and its control(s) are well correlated, as is the case for Upf1 and Cdc53 (Figure S2B). Using multiple controls can further improve classification, as observed for Cdc53 (Figure S3B).

We benchmarked the efficiency of this ratiometric approach by analyzing a reference set of structurally characterized protein pairs and systematically profiling the interactome of Upf1 and Cdc53, Met30, and Irc20. Using NanoBiT ratio thresholds that ensured low false positive rates, we achieved sensitivity levels above 60% for both the reference set of interacting pairs and the best-documented interactors of Upf1 and Cdc53. This sensitivity was comparable to or better than that achieved in previous landmark interactome screens (Figures 1E and 2D). Thus, although based on a limited number of PPIs (35 for our reference set, 7 for Upf1, and 15 for Cdc53), our results suggest that ratiometric NanoBiT experiments are effective for uncovering a broad range of PPIs. In the future, it will be important to determine whether NanoBiT can also successfully profile the interactome of challenging proteins, such as low-abundance or transmembrane proteins.

Despite the high sensitivity of ratiometric NanoBiT, a single putative interactor, Mod5, was identified for Irc20. We did not obtain NanoBiT ratios for histone H4 (Hhf1 and Hhf2) or the cochaperone Ydj1, the only Irc20 interactors reported in at least two independent studies (Table S3), as the corresponding strains did not pass quality controls. Several factors may account for the absence of additional interactors detected by NanoBiT. Since Irc20 exhibits the lowest expression level among the investigated baits, the luminescence signals may be too weak to be detected in our high-throughput experimental setup. The C-terminal LgBiT fragment may also impair Irc20’s interaction with some partners. Additionally, our yeast culture conditions did not promote homologous recombination, which might be necessary for Irc20 to interact with some of its partners.^37^^,^^59^^,^^60^ Further studies are thus needed to unravel Irc20’s interactome and investigate its potential association with Mod5.

In contrast, the interactomes of Cdc53, Met30, and Upf1 revealed multiple interactors, including both well-documented and uncharacterized or unreported partners of these proteins. Many unreported interactors appear biologically relevant, likely revealing previously unexplored aspects of the baits' biology. For instance, although the interaction between Cdc53 and Hel1 has not yet been described, studies in human cells and *C. elegans* demonstrated that Ariadne family RBRs associate and function with cullin-based ubiquitin ligases.^61^^,^^62^^,^^63^ The identification of Hel1 as a Cdc53 interactor suggests that this partnership is conserved in yeast, as proposed elsewhere.^64^ Our data also indicate that Str2 and its paralog YML082W are *bona fide* interactors of Met30. Since these interactions require the C-terminal substrate-interacting domain of Met30 (Figure 3D), Str2 and YML082W may be substrates of the SCF^Met30^ ubiquitin ligase. Alternatively, they could modulate its activity. Overall, the interactomes of Cdc53, Met30, and Upf1 illustrate NanoBiT’s ability to detect diverse interaction types, including both direct and indirect, as well as stable and transient, PPIs.

The ability of ratiometric NanoBiT to detect a broad range of PPIs in live cells under near-endogenous conditions makes it particularly well suited for exploring interactome dynamics under various biological conditions, such as environmental stress or genetic alterations. We illustrated this by examining how mutations in the Upf1 CH domain affect its interactome. We observed, for instance, that Pub1 and Rbs1—two proteins that antagonize NMD-dependent degradation of certain transcripts^65^^,^^66^—relied on an intact CH domain to interact with Upf1, similar to the NMD core proteins and effectors (Figure 4D). This suggests that these proteins may modulate NMD by interfering with the assembly of the NMD core complex or downstream NMD events that depend on the CH domain. Conversely, disrupting the CH domain stimulated Upf1’s interaction with the Dbp2 helicase, a previously described interactor of Upf1^53^^,^^67^^,^^68^ that was not identified in our systematic screen. This indicates that, during the sequential process of NMD, the interaction between Upf1 and Dbp2 likely occurs before the assembly of the NMD core complex. Consequently, Dbp2 may play a role in the identification of NMD targets and/or in NMD activation.

### Limitations of the study

Ratiometric NanoBiT is a versatile method for investigating protein interactomes. One limitation is that it requires tagging bait and prey proteins with LgBiT and SmBiT fragments, which may affect their biological activity and interaction profiles. Notably, the yeast strain libraries we constructed use C-terminal tagging. This is incompatible with the function of certain protein classes, such as tail-anchored proteins. Additionally, the spatial positioning of LgBiT and SmBiT is crucial for reconstituting an active luciferase, and this depends on how the LgBiT and SmBiT fragments are fused to the bait and prey proteins. Thus, employing different versions of the tagged proteins, such as N- and C-terminal fusions, can help achieve more comprehensive interactome coverage.^12^

To benchmark ratiometric NanoBiT, this study focused on soluble proteins localizing to the cytoplasm and the nucleus. Further research is needed to evaluate its performance with proteins that perform poorly in other interactomic assays. Since NanoBiT has already been successfully used to study individual PPIs involving transmembrane proteins,^19^^,^^69^ it would be particularly valuable to determine whether the ratiometric approach described here can effectively profile the interactome of these more challenging proteins.

In any case, our results showcase the effectiveness of ratiometric NanoBiT for interactome studies. This method captures a broad range of both direct and indirect PPIs, providing a complementary approach to generating more comprehensive and high-quality protein interaction maps. Additionally, because NanoBiT assays can probe PPIs in live cells and detect transient interactions, they allow for the exploration of the dynamic properties of interactomes. Ultimately, ratiometric NanoBiT will facilitate the functional investigation of interactomes across different biological contexts, contributing to a deeper understanding of how the organization and dynamics of PPI networks regulate cellular processes.

## Resource availability

### Lead contact

Further information and requests for resources and reagents should be directed to and will be fulfilled by the lead contact, Gwenaël Rabut (gwenael.rabut@univ-rennes.fr).

### Materials availability

Plasmids and yeast strains will be made available by lead contact upon request.

### Data and code availability

• Raw NanoBiT and NanoLuc data, along with analyzed NanoBiT data and literature-curated PPIs, have been deposited at Zenodo and are publicly available at https://doi.org/10.5281/zenodo.13643193, https://doi.org/10.5281/zenodo.13902134, and https://doi.org/10.5281/zenodo.13732204 as of the date of publication.
• The code used to compute NanoBiT ratios has been deposited at Zenodo and is publicly available at https://doi.org/10.5281/zenodo.13644404 as of the date of publication.
• Any additional information required to reanalyze the data reported in this paper is available from the lead contact upon request.

## Acknowledgments

The authors would like to thank Hortense d’Amecourt for her assistance with initial NanoBiT benchmarking experiments. This study was funded by a grant from the National Science Centre, Poland, to N.L. (2021/41/N/NZ2/00551) and by an ANR JCJC grant to G.R. (ANR-16-CE11-0021-01). N.L. received a French Government Scholarship (BGF), a STER scholarship from the Polish National Academic Exchange Agency (NAWA), and a mobility grant from the Région Bretagne. For the purpose of open access, a CC-BY public copyright license has been applied by the authors up to the Author Accepted Manuscript.

## Author contributions

N.L. and G.R. conceptualized the project, designed the study, and obtained funding. N.L., G.L.D., and R.C. performed the investigation. L.R. helped with PPI integration from multiple databases. G.R. analyzed the NanoBiT and NanoLuc data and prepared the figures. N.L. and G.R. wrote the manuscript. G.R. and R.W. supervised the project. M.M. and M.K. prepared and made available the C-SWAT yeast collection. All authors have read and agreed to the published version of the manuscript.

## Declaration of interests

The authors declare no competing interests.

## Declaration of generative AI and AI-assisted technologies in the writing process

During the preparation of this manuscript, the authors used ChatGPT to improve readability and language. The authors reviewed and edited the content as needed and take full responsibility for the content of the publication.

## STAR★Methods

### Key resources table

**Table:** 

REAGENT or RESOURCE	SOURCE	IDENTIFIER
**Chemicals, peptides, and recombinant proteins**		

Canavanine	Sigma-Aldrich	C9758
G418	Formedium	G4185
Fluorescein diacetate	Sigma-Aldrich	F7378
5-Fluorocytosine	Apollo Scientific	PC3735
5-Fluoroorotic acid	Apollo Scientific	PC4054
Furimazine	ChemShuttle	185252
Hygromycin B	ForMedium	HYG5000
Nourseothricin	Werner BioAgents	5.001.000
Thialysine	Bachem	E−1355

**Deposited data**		

Raw NanoBiT and NanoLuc data	This paper	Zenodo: https://doi.org/10.5281/zenodo.13643193
Literature-curated PPIs	This paper	Zenodo: https://doi.org/10.5281/zenodo.13732204
Analyzed NanoBiT data	This paper	Zenodo: https://doi.org/10.5281/zenodo.13902134

**Experimental models:** O**rganisms/strains**		

*S. cerevisiae*: Strain background BY4741 S288c *MATa his3*Δ*1 leu2*Δ*0 met15*Δ*0 ura3*Δ*0*	Brachmann et al.^70^	ATTC: 201388
*S. cerevisiae*: Strain background BY4745 S288c *MATα his3*Δ*1 leu2*Δ*0 met15*Δ*0 ura3*Δ*0*	Brachmann et al.^70^	N/A
*S. cerevisiae*: Strain yMaM1205 BY4745 *can1::STE3pr-LEU2-GAL1pr-NLS-SceI lyp1*Δ	Meurer et al.^23^	N/A
*S. cerevisiae*: Strain scGR1684 BY4741 *fcy1::STE2pr-spHIS5-GAL1pr-NLS-SceI lys2*Δ	This paper	N/A
*S. cerevisiae*: C-SWAT library BY4741 *ORF::C-SWAT-acceptor (L3-CYC1term-ScURA3-hph*Δ*N-ALG9term-L4)*	Meurer et al.^23^	N/A
*S. cerevisiae*: LgBiT library BY4745 *ORF::LgBiT-10HIS-HPH can1*Δ*::STE3pr-LEU2-GAL1pr-NLS-SceI lyp1*Δ	Le Boulch et al.^27^	N/A
*S. cerevisiae*: SmBiT library BY4741 *ORF::SmBiT-NAT fcy1*Δ*::STE2pr-spHIS5-GAL1pr-NLS-SceI*	This paper	N/A
*S. cerevisiae*: NanoLuc library BY4745 *ORF::NanoLuc-HPH can1*Δ*::STE3pr-LEU2-GAL1pr-NLS-SceI lyp1*Δ	This paper	N/A
*S. cerevisiae*: Strain scGLD0263 BY4741 *upf1(*Δ*62-152)::SmBiT-NAT*	This paper	N/A
*S. cerevisiae*: Strain scGLD0264 BY4741 *upf1(C122S)::SmBiT-NAT*	This paper	N/A
*S. cerevisiae*: Strain scGLD0265 BY4741 *upf1(Y64A)::SmBiT-NAT*	This paper	N/A
*S. cerevisiae*: Strain scGLD0266 BY4741 *upf1(I67A)::SmBiT-NAT*	This paper	N/A
*S. cerevisiae*: Strain scGLD0267 BY4741 *upf1(*Δ*195-208)::SmBiT-NAT*	This paper	N/A
*S. cerevisiae*: Strain scGLD0268 BY4741 *upf1(E202K,W205K)::SmBiT-NAT*	This paper	N/A
*S. cerevisiae*: Strain scGLD0319 BY4745 *MET4::LgBiT-10HIS-HPH 3HA-MET30::SmBiT-NAT met32*Δ*::URA3 can1*Δ*::STE3pr-LEU2-GAL1pr-NLS-SceI lyp1*Δ	This paper	N/A
*S. cerevisiae*: Strain scGLD0367 BY4745 *MET4::LgBiT-10HIS-HPH 3HA-met30(1–227)::SmBiT-NAT met32*Δ*::URA3 can1*Δ*::STE3pr-LEU2-GAL1pr-NLS-SceI lyp1*Δ	This paper	N/A
*S. cerevisiae*: Strain scGLD0321 BY4745 *MET28::LgBiT-10HIS-HPH 3HA-MET30::SmBiT-NAT met32*Δ*::URA3 can1*Δ*::STE3pr-LEU2-GAL1pr-NLS-SceI lyp1*Δ	This paper	N/A
*S. cerevisiae*: Strain scGLD0369 BY4745 *MET28::LgBiT-10HIS-HPH 3HA-met30(1–227)::SmBiT-NAT met32*Δ*::URA3 can1*Δ*::STE3pr-LEU2-GAL1pr-NLS-SceI lyp1*Δ	This paper	N/A
*S. cerevisiae*: Strain scGLD0318 BY4745 *MET31::LgBiT-10HIS-HPH 3HA-MET30::SmBiT-NAT met32*Δ*::URA3 can1*Δ*::STE3pr-LEU2-GAL1pr-NLS-SceI lyp1*Δ	This paper	N/A
*S. cerevisiae*: Strain scGLD0366 BY4745 *MET31::LgBiT-10HIS-HPH 3HA met30(1–227)::SmBiT-NAT met32*Δ*::URA3 can1*Δ*::STE3pr-LEU2-GAL1pr-NLS-SceI lyp1*Δ	This paper	N/A
*S. cerevisiae*: Strain scGLD0316 BY4745 *STR2::LgBiT-10HIS-HPH 3HA-MET30::SmBiT-NAT met32*Δ*::URA3 can1*Δ*::STE3pr-LEU2-GAL1pr-NLS-SceI lyp1*Δ	This paper	N/A
*S. cerevisiae*: Strain scGLD0376 BY4745 *STR2::LgBiT-10HIS-HPH 3HA-met30(1–227)::SmBiT-NAT met32*Δ*::URA3 can1*Δ*::STE3pr-LEU2-GAL1pr-NLS-SceI lyp1*Δ	This paper	N/A
*S. cerevisiae*: Strain scGLD0317 BY4745 *YML082W::LgBiT-10HIS-HPH 3HA-MET30::SmBiT-NAT met32*Δ*::URA3 can1*Δ*::STE3pr-LEU2-GAL1pr-NLS-SceI lyp1*Δ	This paper	N/A
*S. cerevisiae*: Strain scGLD0365 BY4745 *YML082W::LgBiT-10HIS-HPH 3HA-met30(1–227)::SmBiT-NAT met32*Δ*::URA3 can1*Δ*::STE3pr-LEU2-GAL1pr-NLS-SceI lyp1*Δ	This paper	N/A

**Recombinant DNA**		

Plasmid pAB0006: pSD-C3v2_yNanoLuc	This paper	N/A
Plasmid pGR0938: pSD-C4v2_SmBiT	This paper	N/A

**Software and algorithms**		

R software (version 4.1.3)	R Foundation for Statistical Computing	https://www.r-project.org/
RStudio (version 2023.12.1.402)	R Foundation for Statistical Computing	https://rstudio.com/
ggplot2 R package (version 3.5.1)	Wickham^71^	https://ggplot2.tidyverse.org/
corrmorant R package (version 0.0.0.9007)	Roman	https://github.com/r-link/corrmorant
clusterProfiler R package (version 4.22.0)	Yu et al.^72^	https://doi.org/10.18129/B9.bioc.clusterProfiler
org.Sc.sgd.db R package (version 3.14.0)	Carlson	https://doi.org/10.18129/B9.bioc.org.Sc.sgd.db
R code for NanoBiT data analysis	This paper	Zenodo: https://doi.org/10.5281/zenodo.13644404

### Experimental model and study participant details

All yeast strains used in this study are listed in the key resources table and are isogenic derivatives of BY4741 and BY4745.^70^ Genome modifications (chromosomal gene tagging and editing) of individual strains were carried out using conventional procedures based on PCR targeting and plasmid integration. All experiments were performed at 30°C in synthetic complete (SC) medium (1.7 g/L yeast nitrogen base without amino acids and ammonium sulfate, 2 g/L amino acid mix) with glucose as a carbon source (20 g/L) and monosodium glutamate (MSG) as a nitrogen source (1 g/L), unless stated otherwise.

### Method details

#### Construction of genome-wide NanoBiT and NanoLuc yeast strain libraries

The SWAP-Tag (SWAT) method was used to assemble genome-wide libraries of haploid yeast strains expressing proteins C-terminally fused to LgBiT-His, SmBiT or NanoLuc tags. The procedure described by Meurer et al.^23^ was followed to construct the *MAT*alpha LgBiT library, as previously reported.^27^ The same procedure was used to construct the *MAT*alpha NanoLuc library. Briefly, the plasmid pAB0006 (which provides a donor module containing the sequence of NanoLuc followed by a heterologous terminator and a truncated Hygromycin B resistance cassette) was transformed into the yMAM1205 strain. The transformed strain was crossed with the full C-SWAT library arrayed in a 384-colony format. The colonies were sequentially pinned on appropriate media to select diploids, sporulate, select haploids, recombine the acceptor and donor module, and select the recombined strains (see Table S1 for the media used at each step).

This procedure was adapted to construct the *MAT*a SmBiT collection. We first constructed the scGR1684 strain in which the *FCY1* gene was replaced by the *STE2pr-spHIS5-GAL1pr-NLS-SceI* cassette. *FCY1* encodes cytosine deaminase. Its deletion confers resistance to 5-fluorocytosine (5-FC) (in wild type cells, 5-FC is deaminated by cytosine deaminase to produce 5-fluorouracil, which causes RNA miscoding).^73^ This marker is used to counterselect diploids during haploid selection steps. *STE2pr-spHIS5-GAL1pr-NLS-SceI* enables both selection of *MAT*a haploids (the *STE2pr-spHIS5* marker is only expressed in *MAT*a cells) and conditional expression of the I-*Sce*I endonuclease (to induce recombination between the acceptor and donor modules). This strain was transformed with plasmid pGR0938, which provides an SWAT donor module containing the sequence of SmBiT followed by a heterologous terminator and a Nourseothricin (clonNAT) resistance cassette. The transformed strain was crossed with the full C-SWAT library arrayed in a 384-colony format and the colonies were sequentially pinned on appropriate media (Table S1).

To construct the NanoBiT strains, individual strains expressing Cdc53-SmBiT, Irc20-SmBiT, Met30-SmBiT, and Upf1-SmBiT were isolated from the SmBiT library. These strains were crossed with the entire LgBiT-His library previously arrayed in a 384-colony format. The *MA*Talpha haploid progeny was selected using appropriate media (Table S1) to obtain collections of Cdc53, Irc20, Met30 and Upf1 NanoBiT strains.

Manipulations of yeast colonies for the constructions of all libraries were performed using a microbial pinning robot (ROTOR HDA, Singer Instruments).

#### NanoBiT and NanoLuc assays

For low-throughput NanoBiT and NanoLuc assays, yeast strains were distributed in transparent 96-well microplates containing 125 μL of SC(MSG) medium and grown overnight at 30°C. The overnight cultures were diluted ten times in 125 μL of SC(MSG), further grown for 3 h at 30°C, and the optical density (OD_600_) was measured. 20 μL of fresh cultures were then transferred to white 96-well half-area microtiter plates (Greiner Bio-One) previously filled with 20 μL/well of SC(MSG) containing 100 μM furimazine (ChemShuttle). The microplates were then incubated for 10 min in the dark before luminescence signals were recorded. Luminescence measurements were performed for 1 s per well at a distance of 0.1 mm between the plate and the detector.

For genome-wide NanoBiT and NanoLuc assays, yeast strains arrayed in a 1536-colony format were freshly deposited on SC(MSG) plates and grown overnight at 25°C. The colonies were then transferred into four white 384-well shallow microplates (ProxiPlate Plus, PerkinElmer) previously filled with 20 μL/well of SC(MSG) containing 50 μM furimazine (ChemShuttle) and 10 μM fluorescein diacetate (Sigma Aldrich). The microplates were then incubated for 10 min in the dark before fluorescence and luminescence signals were recorded. Fluorescence measurements were performed using an excitation wavelength of 500 nm and an emission wavelength of 520 nm with 100 flashes at 13 mm height. Luminescence measurements were performed for 1 s per well at a distance of 0.1 mm between the plate and the detector.

Fluorescence, luminescence and OD_600_ measurements were all performed at room temperature with a multimode microplate reader (Ensight, PerkinElmer).

### Quantification and statistical analysis

#### Analysis of the NanoBiT and NanoLuc measurements

The raw luminescence measurements were background subtracted, corrected by the corresponding background-subtracted cell density measurements (fluorescence or OD_600_ depending on the experiment) and log2 transformed. To correct for day-to-day and plate-to-plate variations in luminescence intensities, each log2 transformed luminescence signal was plotted against the median of the corresponding signals from all experimental replicates. Linear regressions were performed for each plate and the best fit served to adjust the individual log2 transformed luminescence signals. The obtained values were then used to compute mean NanoBiT and NanoLuc signals as well as NanoBiT ratios. All data processing and analysis were performed using R. Results were presented using the ggplot2^71^ and corrmorant packages.

#### Classification of systematic NanoBiT data

Literature-curated interactors of Cdc53 and Upf1 were retrieved from the BioGRID, DIP, IntAct, and MINT databases using the PSICQUIC service^74^ on March 20, 2024 (BiogGRID release 4.4.231 and IntAct release 2024-02-14 containing data from IntAct, DIP and MINT). Experimental observations recorded in the various databases were filtered to exclude entries that did not correspond to PPIs (e.g., genetic interactions and protein-nucleic acid interactions). Explicit and implicit redundancies between entries from the different databases were detected and eliminated by taking into account the ancestor-descendent relationship of the molecular interaction terms used to describe each experimental observation, as previously described.^75^ Cdc53 and Upf1 interactors supported by three or more experimental pieces of evidence were considered as positives and used to enumerate true positives (TP) and false negatives (FN) across varying NanoBiT ratios.

Unlike positive interactors, it is not possible to list proteins that do not interact with Cdc53 or Upf1 with absolute certainty. Therefore, we assembled lists of proteins without recorded interaction data for Cdc53 or Upf1 in the BiGRID, DIP, IntAct, and MINT databases. These proteins were further filtered to exclude those annotated with GO terms related to Cdc53 or Upf1 activities. The terms used for this filtering were ‘ubiquitin-dependent protein catabolic process’ (GO:0006511) and ‘protein ubiquitination’ (GO:0016567) for Cdc53, and ‘mRNA catabolic process’ (GO:0006402) and ‘translation’ (GO:0006412) for Upf1. The vast majority of the remaining proteins are expected not to interact with Cdc53 or Upf1 and were therefore used to estimate true negatives (TN) and false positives (FP) across varying NanoBiT ratios.

The TP, FN, TN and FP then served to compute the classification sensitivity, precision, specificity and Matthews correlation coefficient (MCC) at each ratio using the following equations:

$$Sensitivity=\frac{TP}{TP+FN}$$

$$Precision=\frac{TP}{TP+FP}$$

$$Specificity=\frac{TN}{TN+FP}$$

$$MCC=\frac{TP\cdot TN-FP\cdot FN}{\sqrt{\left(TP+FP\right)\cdot \left(TP+FN\right)\cdot \left(TN+FP\right)\cdot \left(TN+FN\right)}}$$

Classification performance was compared by plotting receiver operating characteristic (ROC, sensitivity vs. 1-specificity) and precision-recall (PR, precision vs. sensitivity) curves, and by quantifying the area under these curves. The Matthews correlation coefficient (MCC) was used to identify the optimal classification threshold that maximizes both precision and sensitivity.

#### GO term enrichment analysis

Gene Ontology (GO) term enrichment analysis was conducted on the identified interactors of Cdc53, Met30 and Upf1 using the R packages clusterProfiler^72^ and org.Sc.sgd.db. Enrichment *p*-values were calculated using as background all prey proteins for which a NanoBiT ratio was determined and adjusted for multiple hypotheses testing using the Benjamini–Hochberg method.^76^ All GO terms significantly enriched with an adjusted *p*-value <0.01 are reported in Table S4.

## Supplemental information

Document S1. Figures S1–S4 and Table S1

Table S2. Reference protein pairs investigated in this study, related to Figure 1

Table S3. Literature-curated and NanoBiT-detected interactors of Upf1, Cdc53, Met30, and Irc20, related to Figures 2, 3, and 4

Table S4. Results of GO term enrichment analysis for NanoBiT-detected interactors of Upf1, Cdc53, and Met30, related to Figure S4

Document S2. Article plus supplemental information

## References

[1] Vidal M., Cusick M.E., Barabási A.-L. (2011). Interactome networks and human disease. Cell.

[2] Cheng F., Zhao J., Wang Y., Lu W., Liu Z., Zhou Y., Martin W.R., Wang R., Huang J., Hao T. (2021). Comprehensive characterization of protein-protein interactions perturbed by disease mutations. Nat. Genet..

[3] Uetz P., Giot L., Cagney G., Mansfield T.A., Judson R.S., Knight J.R., Lockshon D., Narayan V., Srinivasan M., Pochart P. (2000). A comprehensive analysis of protein-protein interactions in Saccharomyces cerevisiae. Nature.

[4] Stelzl U., Worm U., Lalowski M., Haenig C., Brembeck F.H., Goehler H., Stroedicke M., Zenkner M., Schoenherr A., Koeppen S. (2005). A human protein-protein interaction network: a resource for annotating the proteome. Cell.

[5] Gavin A.-C., Aloy P., Grandi P., Krause R., Boesche M., Marzioch M., Rau C., Jensen L.J., Bastuck S., Dümpelfeld B. (2006). Proteome survey reveals modularity of the yeast cell machinery. Nature.

[6] Tarassov K., Messier V., Landry C.R., Radinovic S., Serna Molina M.M., Shames I., Malitskaya Y., Vogel J., Bussey H., Michnick S.W. (2008). An in vivo map of the yeast protein interactome. Science.

[7] Rolland T., Taşan M., Charloteaux B., Pevzner S.J., Zhong Q., Sahni N., Yi S., Lemmens I., Fontanillo C., Mosca R. (2014). A proteome-scale map of the human interactome network. Cell.

[8] Huttlin E.L., Ting L., Bruckner R.J., Gebreab F., Gygi M.P., Szpyt J., Tam S., Zarraga G., Colby G., Baltier K. (2015). The BioPlex Network: A Systematic Exploration of the Human Interactome. Cell.

[9] Vo T.V., Das J., Meyer M.J., Cordero N.A., Akturk N., Wei X., Fair B.J., Degatano A.G., Fragoza R., Liu L.G. (2016). A Proteome-wide Fission Yeast Interactome Reveals Network Evolution Principles from Yeasts to Human. Cell.

[10] Luck K., Kim D.-K., Lambourne L., Spirohn K., Begg B.E., Bian W., Brignall R., Cafarelli T., Campos-Laborie F.J., Charloteaux B. (2020). A reference map of the human binary protein interactome. Nature.

[11] Tang H.-W., Spirohn K., Hu Y., Hao T., Kovács I.A., Gao Y., Binari R., Yang-Zhou D., Wan K.H., Bader J.S. (2023). Next-generation large-scale binary protein interaction network for Drosophila melanogaster. Nat. Commun..

[12] Choi S.G., Olivet J., Cassonnet P., Vidalain P.-O., Luck K., Lambourne L., Spirohn K., Lemmens I., Dos Santos M., Demeret C. (2019). Maximizing binary interactome mapping with a minimal number of assays. Nat. Commun..

[13] Hao B., Kovács I.A. (2023). A positive statistical benchmark to assess network agreement. Nat. Commun..

[14] Venkatesan K., Rual J.-F., Vazquez A., Stelzl U., Lemmens I., Hirozane-Kishikawa T., Hao T., Zenkner M., Xin X., Goh K.-I. (2009). An empirical framework for binary interactome mapping. Nat. Methods.

[15] Michnick S.W., Ear P.H., Manderson E.N., Remy I., Stefan E. (2007). Universal strategies in research and drug discovery based on protein-fragment complementation assays. Nat. Rev. Drug Discov..

[16] Blaszczak E., Lazarewicz N., Sudevan A., Wysocki R., Rabut G. (2021). Protein-fragment complementation assays for large-scale analysis of protein-protein interactions. Biochem. Soc. Trans..

[17] Pelletier J.N., Campbell-Valois F.X., Michnick S.W. (1998). Oligomerization domain-directed reassembly of active dihydrofolate reductase from rationally designed fragments. Proc. Natl. Acad. Sci. USA.

[18] Hu C.-D., Chinenov Y., Kerppola T.K. (2002). Visualization of interactions among bZIP and Rel family proteins in living cells using bimolecular fluorescence complementation. Mol. Cell.

[19] Dixon A.S., Schwinn M.K., Hall M.P., Zimmerman K., Otto P., Lubben T.H., Butler B.L., Binkowski B.F., Machleidt T., Kirkland T.A. (2016). NanoLuc Complementation Reporter Optimized for Accurate Measurement of Protein Interactions in Cells. ACS Chem. Biol..

[20] Remy I., Michnick S.W. (2006). A highly sensitive protein-protein interaction assay based on Gaussia luciferase. Nat. Methods.

[21] Tebo A.G., Gautier A. (2019). A split fluorescent reporter with rapid and reversible complementation. Nat. Commun..

[22] Hall M.P., Unch J., Binkowski B.F., Valley M.P., Butler B.L., Wood M.G., Otto P., Zimmerman K., Vidugiris G., Machleidt T. (2012). Engineered luciferase reporter from a deep sea shrimp utilizing a novel imidazopyrazinone substrate. ACS Chem. Biol..

[23] Meurer M., Duan Y., Sass E., Kats I., Herbst K., Buchmuller B.C., Dederer V., Huber F., Kirrmaier D., Štefl M. (2018). Genome-wide C-SWAT library for high-throughput yeast genome tagging. Nat. Methods.

[24] Ho Y., Gruhler A., Heilbut A., Bader G.D., Moore L., Adams S.-L., Millar A., Taylor P., Bennett K., Boutilier K. (2002). Systematic identification of protein complexes in Saccharomyces cerevisiae by mass spectrometry. Nature.

[25] Krogan N.J., Cagney G., Yu H., Zhong G., Guo X., Ignatchenko A., Li J., Pu S., Datta N., Tikuisis A.P. (2006). Global landscape of protein complexes in the yeast Saccharomyces cerevisiae. Nature.

[26] Michaelis A.C., Brunner A.-D., Zwiebel M., Meier F., Strauss M.T., Bludau I., Mann M. (2023). The social and structural architecture of the yeast protein interactome. Nature.

[27] Le Boulch M., Brossard A., Le Dez G., Léon S., Rabut G. (2020). Sensitive detection of protein ubiquitylation using a protein fragment complementation assay. J. Cell Sci..

[28] Murphy R., Wente S.R. (1996). An RNA-export mediator with an essential nuclear export signal. Nature.

[29] Güttler T., Görlich D. (2011). Ran-dependent nuclear export mediators: a structural perspective. EMBO J..

[30] Braun P., Tasan M., Dreze M., Barrios-Rodiles M., Lemmens I., Yu H., Sahalie J.M., Murray R.R., Roncari L., de Smet A.-S. (2009). An experimentally derived confidence score for binary protein-protein interactions. Nat. Methods.

[31] Leeds P., Peltz S.W., Jacobson A., Culbertson M.R. (1991). The product of the yeast UPF1 gene is required for rapid turnover of mRNAs containing a premature translational termination codon. Genes Dev..

[32] He F., Brown A.H., Jacobson A. (1997). Upf1p, Nmd2p, and Upf3p are interacting components of the yeast nonsense-mediated mRNA decay pathway. Mol. Cell Biol..

[33] Feldman R.M., Correll C.C., Kaplan K.B., Deshaies R.J. (1997). A complex of Cdc4p, Skp1p, and Cdc53p/cullin catalyzes ubiquitination of the phosphorylated CDK inhibitor Sic1p. Cell.

[34] Skowyra D., Craig K.L., Tyers M., Elledge S.J., Harper J.W. (1997). F-box proteins are receptors that recruit phosphorylated substrates to the SCF ubiquitin-ligase complex. Cell.

[35] Rouillon A., Barbey R., Patton E.E., Tyers M., Thomas D. (2000). Feedback-regulated degradation of the transcriptional activator Met4 is triggered by the SCF(Met30 )complex. EMBO J..

[36] Flick K., Ouni I., Wohlschlegel J.A., Capati C., McDonald W.H., Yates J.R., Kaiser P. (2004). Proteolysis-independent regulation of the transcription factor Met4 by a single Lys 48-linked ubiquitin chain. Nat. Cell Biol..

[37] Alvaro D., Lisby M., Rothstein R. (2007). Genome-wide analysis of Rad52 foci reveals diverse mechanisms impacting recombination. PLoS Genet..

[38] Richardson A., Gardner R.G., Prelich G. (2013). Physical and genetic associations of the Irc20 ubiquitin ligase with Cdc48 and SUMO. PLoS One.

[39] Willems A.R., Lanker S., Patton E.E., Craig K.L., Nason T.F., Mathias N., Kobayashi R., Wittenberg C., Tyers M. (1996). Cdc53 targets phosphorylated G1 cyclins for degradation by the ubiquitin proteolytic pathway. Cell.

[40] Mathias N., Johnson S.L., Winey M., Adams A.E., Goetsch L., Pringle J.R., Byers B., Goebl M.G. (1996). Cdc53p acts in concert with Cdc4p and Cdc34p to control the G1-to-S-phase transition and identifies a conserved family of proteins. Mol. Cell Biol..

[41] Takahashi S., Araki Y., Ohya Y., Sakuno T., Hoshino S.-I., Kontani K., Nishina H., Katada T. (2008). Upf1 potentially serves as a RING-related E3 ubiquitin ligase via its association with Upf3 in yeast. RNA.

[42] Chicco D., Jurman G. (2020). The advantages of the Matthews correlation coefficient (MCC) over F1 score and accuracy in binary classification evaluation. BMC Genom..

[43] Ouni I., Flick K., Kaiser P. (2010). A transcriptional activator is part of an SCF ubiquitin ligase to control degradation of its cofactors. Mol. Cell.

[44] Hansen J., Johannesen P.F. (2000). Cysteine is essential for transcriptional regulation of the sulfur assimilation genes in Saccharomyces cerevisiae. Mol. Gen. Genet..

[45] Yu H., Braun P., Yildirim M.A., Lemmens I., Venkatesan K., Sahalie J., Hirozane-Kishikawa T., Gebreab F., Li N., Simonis N. (2008). High-quality binary protein interaction map of the yeast interactome network. Science.

[46] Kitagawa K., Skowyra D., Elledge S.J., Harper J.W., Hieter P. (1999). SGT1 encodes an essential component of the yeast kinetochore assembly pathway and a novel subunit of the SCF ubiquitin ligase complex. Mol. Cell.

[47] Hiser L., Basson M.E., Rine J. (1994). ERG10 from Saccharomyces cerevisiae encodes acetoacetyl-CoA thiolase. J. Biol. Chem..

[48] Dehecq M., Decourty L., Namane A., Proux C., Kanaan J., Le Hir H., Jacquier A., Saveanu C. (2018). Nonsense-mediated mRNA decay involves two distinct Upf1-bound complexes. EMBO J..

[49] Collart M.A. (2016). The Ccr4-Not complex is a key regulator of eukaryotic gene expression. Wiley Interdiscip. Rev. RNA.

[50] Tharun S. (2009). Lsm1-7-Pat1 complex: a link between 3’ and 5'-ends in mRNA decay?. RNA Biol..

[51] Sharif H., Conti E. (2013). Architecture of the Lsm1-7-Pat1 complex: a conserved assembly in eukaryotic mRNA turnover. Cell Rep..

[52] He F., Ganesan R., Jacobson A. (2013). Intra- and intermolecular regulatory interactions in Upf1, the RNA helicase central to nonsense-mediated mRNA decay in yeast. Mol. Cell Biol..

[53] He F., Jacobson A. (1995). Identification of a novel component of the nonsense-mediated mRNA decay pathway by use of an interacting protein screen. Genes Dev..

[54] Min E.E., Roy B., Amrani N., He F., Jacobson A. (2013). Yeast Upf1 CH domain interacts with Rps26 of the 40S ribosomal subunit. RNA.

[55] He F., Jacobson A. (2015). Nonsense-Mediated mRNA Decay: Degradation of Defective Transcripts Is Only Part of the Story. Annu. Rev. Genet..

[56] Kadlec J., Guilligay D., Ravelli R.B., Cusack S. (2006). Crystal structure of the UPF2-interacting domain of nonsense-mediated mRNA decay factor UPF1. RNA.

[57] Choi H., Larsen B., Lin Z.-Y., Breitkreutz A., Mellacheruvu D., Fermin D., Qin Z.S., Tyers M., Gingras A.-C., Nesvizhskii A.I. (2011). SAINT: probabilistic scoring of affinity purification-mass spectrometry data. Nat. Methods.

[58] Pu S., Vlasblom J., Turinsky A., Marcon E., Phanse S., Trimble S.S., Olsen J., Greenblatt J., Emili A., Wodak S.J. (2015). Extracting high confidence protein interactions from affinity purification data: at the crossroads. J. Proteomics.

[59] Miura T., Yamana Y., Usui T., Ogawa H.I., Yamamoto M.-T., Kusano K. (2012). Homologous recombination via synthesis-dependent strand annealing in yeast requires the Irc20 and Srs2 DNA helicases. Genetics.

[60] Jalal D., Chalissery J., Iqbal M., Hassan A.H. (2021). The ATPase Irc20 facilitates Rad51 chromatin enrichment during homologous recombination in yeast Saccharomyces cerevisiae. DNA Repair.

[61] Scott D.C., Rhee D.Y., Duda D.M., Kelsall I.R., Olszewski J.L., Paulo J.A., de Jong A., Ovaa H., Alpi A.F., Harper J.W., Schulman B.A. (2016). Two Distinct Types of E3 Ligases Work in Unison to Regulate Substrate Ubiquitylation. Cell.

[62] Dove K.K., Kemp H.A., Di Bona K.R., Reiter K.H., Milburn L.J., Camacho D., Fay D.S., Miller D.L., Klevit R.E. (2017). Two functionally distinct E2/E3 pairs coordinate sequential ubiquitination of a common substrate in Caenorhabditis elegans development. Proc. Natl. Acad. Sci. USA.

[63] Hüttenhain R., Xu J., Burton L.A., Gordon D.E., Hultquist J.F., Johnson J.R., Satkamp L., Hiatt J., Rhee D.Y., Baek K. (2019). ARIH2 Is a Vif-Dependent Regulator of CUL5-Mediated APOBEC3G Degradation in HIV Infection. Cell Host Microbe.

[64] Kong K.-Y.E., Fischer B., Meurer M., Kats I., Li Z., Rühle F., Barry J.D., Kirrmaier D., Chevyreva V., San Luis B.-J. (2021). Timer-based proteomic profiling of the ubiquitin-proteasome system reveals a substrate receptor of the GID ubiquitin ligase. Mol. Cell.

[65] Ruiz-Echevarría M.J., Peltz S.W. (2000). The RNA binding protein Pub1 modulates the stability of transcripts containing upstream open reading frames. Cell.

[66] Cieśla M., Turowski T.W., Nowotny M., Tollervey D., Boguta M. (2020). The expression of Rpb10, a small subunit common to RNA polymerases, is modulated by the R3H domain-containing Rbs1 protein and the Upf1 helicase. Nucleic Acids Res..

[67] Bond A.T., Mangus D.A., He F., Jacobson A. (2001). Absence of Dbp2p alters both nonsense-mediated mRNA decay and rRNA processing. Mol. Cell Biol..

[68] Wery M., Descrimes M., Vogt N., Dallongeville A.-S., Gautheret D., Morillon A. (2016). Nonsense-Mediated Decay Restricts LncRNA Levels in Yeast Unless Blocked by Double-Stranded RNA Structure. Mol. Cell.

[69] Inoue A., Raimondi F., Kadji F.M.N., Singh G., Kishi T., Uwamizu A., Ono Y., Shinjo Y., Ishida S., Arang N. (2019). Illuminating G-Protein-Coupling Selectivity of GPCRs. Cell.

[70] Brachmann C.B., Davies A., Cost G.J., Caputo E., Li J., Hieter P., Boeke J.D. (1998). Designer deletion strains derived from Saccharomyces cerevisiae S288C: a useful set of strains and plasmids for PCR-mediated gene disruption and other applications. Yeast.

[71] Wickham H. (2016).

[72] Yu G., Wang L.-G., Han Y., He Q.-Y. (2012). clusterProfiler: an R package for comparing biological themes among gene clusters. OMICS.

[73] Hartzog P.E., Nicholson B.P., McCusker J.H. (2005). Cytosine deaminase MX cassettes as positive/negative selectable markers in Saccharomyces cerevisiae. Yeast.

[74] del-Toro N., Dumousseau M., Orchard S., Jimenez R.C., Galeota E., Launay G., Goll J., Breuer K., Ono K., Salwinski L., Hermjakob H. (2013). A new reference implementation of the PSICQUIC web service. Nucleic Acids Res..

[75] Melkonian M., Juigné C., Dameron O., Rabut G., Becker E. (2022). Towards a reproducible interactome: semantic-based detection of redundancies to unify protein-protein interaction databases. Bioinformatics.

[76] Benjamini Y., Hochberg Y. (1995). Controlling the false discovery rate: A practical and powerful approach to multiple testing. J. R. Stat. Soc. Series B Stat. Methodol..

