# Supplementary material for: Accurate and sensitive interactome profiling using a quantitative protein-fragment complementation assay
Source: Cell Rep Methods. 2024 Oct 21;4(10):100880. doi: 10.1016/j.crmeth.2024.100880 (PMC11573789; doi:10.1016/j.crmeth.2024.100880)
Supplement: Document S1. Figures S1–S4 and Table S1 [file mmc1.pdf]

Cell Reports Methods, Volume *4*

Supplemental information

Accurate and sensitive interactome profiling using a quantitative protein-fragment complementation assay

Natalia Lazarewicz, Gaëlle Le Dez, Romina Cerjani, Lunelys Runeshaw, Matthias Meurer, Michael Knop, Robert Wysocki, and Gwenaël Rabut

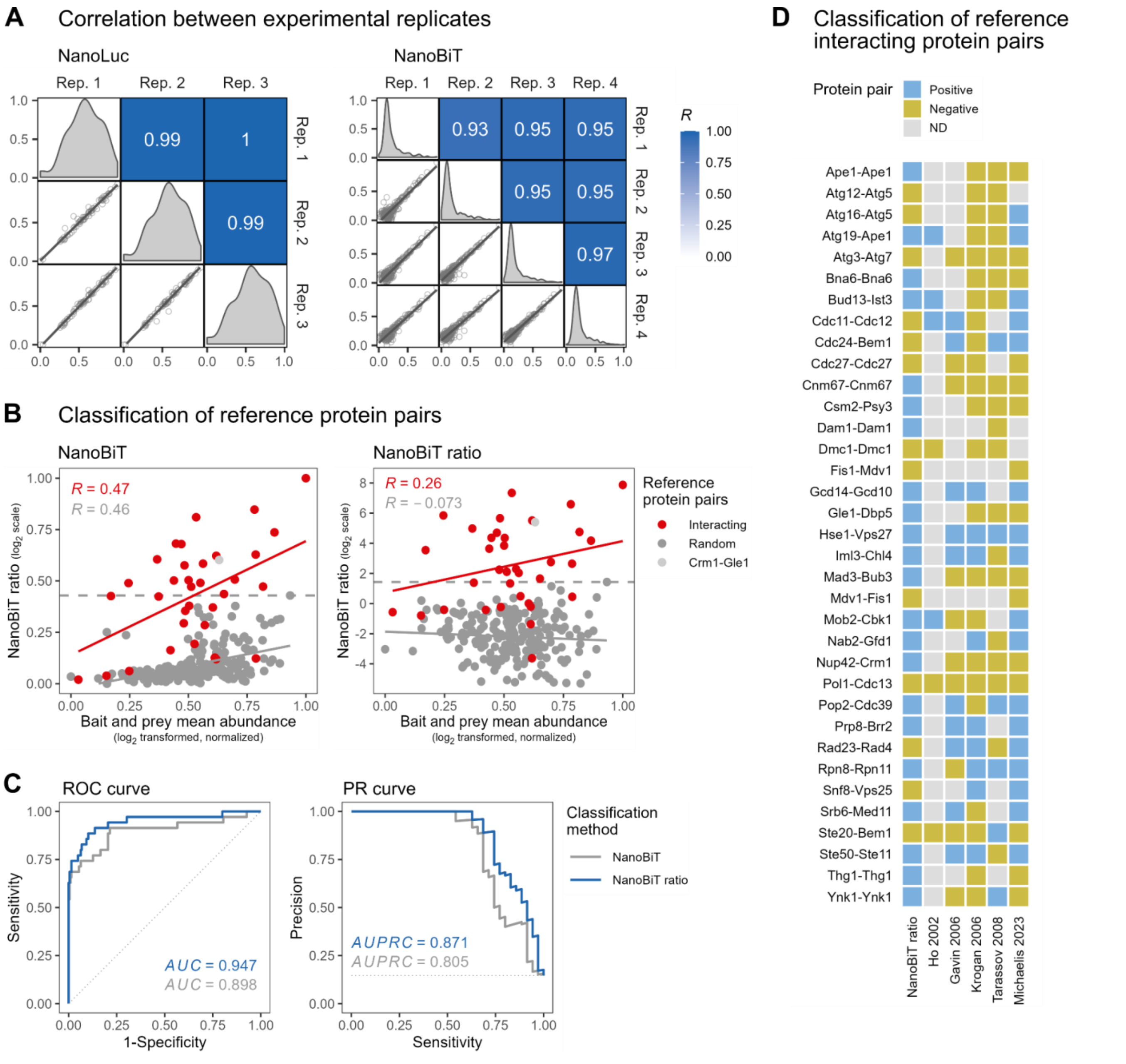

Figure S1. NanoBiT data for reference protein pairs, related to Figure 1.

A) Reproducibility of NanoLuc and NanoBiT measurements. Matrices presenting the correlation among replicates of luminescence measurements for NanoLuc and NanoBiT strains of reference protein pairs. The plots in the lower triangle of each matrix depict individual luminescence measurements for each replicate against the other replicates. Linear regressions are shown in gray. The plots in the diagonal of each matrix depict data densities for each replicate. The heatmap in the upper triangle of each matrix depicts color-coded Pearson correlation coefficients (*R*).

B) Comparison of NanoBiT and NanoBiT ratio data. Scatter plots showing the NanoBiT signal (left) or NanoBiT ratio (right) plotted against the mean NanoLuc intensities for each protein pair. Data are from four experimental replicates for NanoBiT and three for NanoLuc. Interacting pairs are shown in red, while random, likely non-interacting pairs are shown in gray. Linear regressions are displayed with the same colors. The Crm1-Gle1 pair is shown in light gray. The horizontal dashed lines indicate the highest NanoBiT or NanoBiT ratio value observed among random pairs, excluding the Crm1-Gle1 pair.

C) Performance comparison of NanoBiT and NanoBiT ratio classifiers. Receiver operating characteristic (ROC) and precision-recall (PR) curves are shown for classifiers using NanoBiT signals (gray) or NanoBiT ratios (blue). The areas under the ROC curves (*AUC*) and the areas under the PR curves (*AUPRC*) are indicated in the same colors.

D) Comparison of the reference interacting pairs detected by NanoBiT and in large-scale interactome studies. Positive pairs are indicated in light blue, negative pairs are in yellow and not determined (ND) pairs in gray. The interactome studies are: Ho et al. [S1], Gavin et al. [S2], Krogan et al. [S3], Tarassov et al. [S4], and Michaelis et al. [S5].

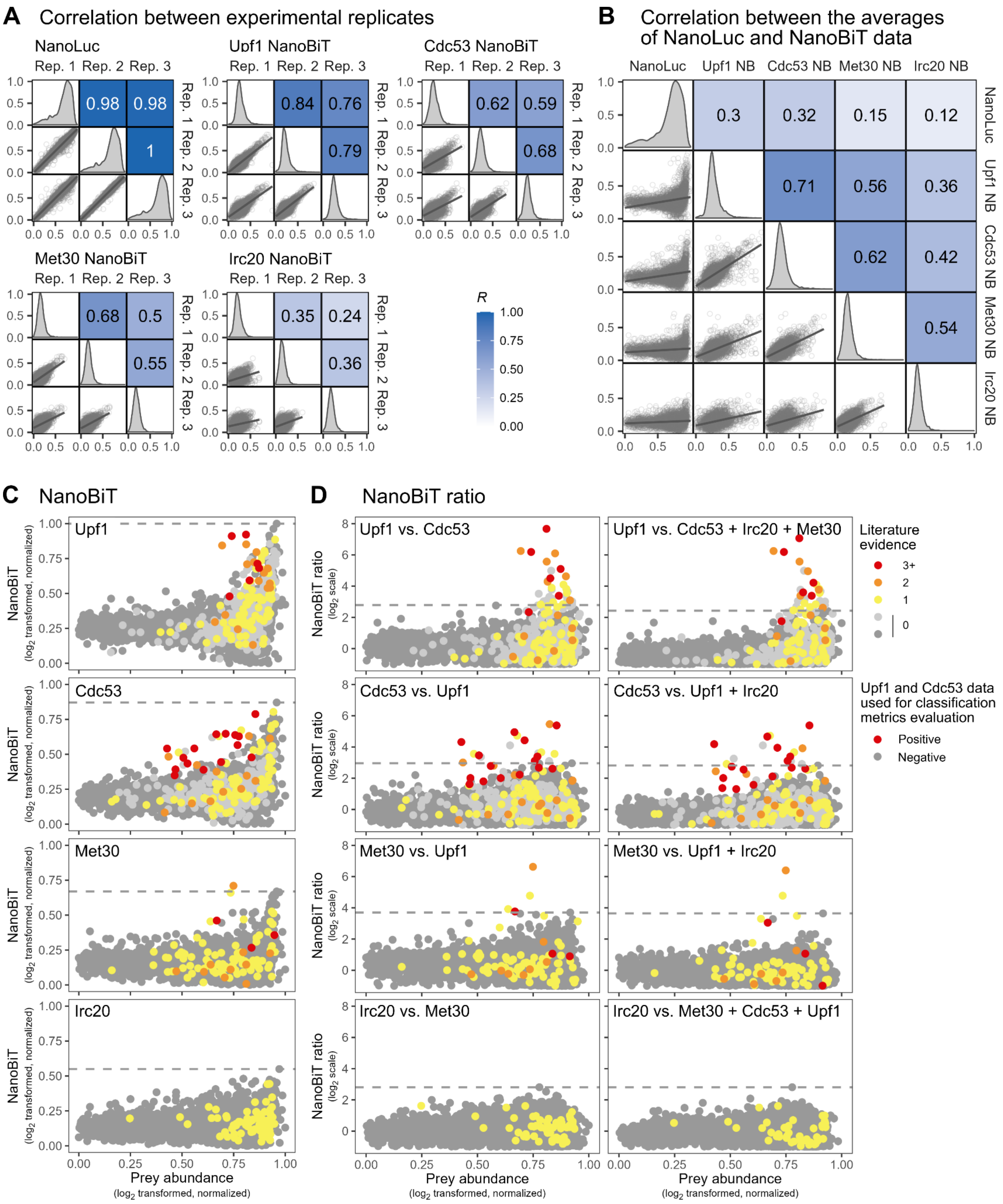

Figure S2. NanoBiT data for Upf1, Cdc53, Met30 and Irc20, related to Figure 2.

A) Reproducibility of NanoLuc and NanoBiT measurements. Matrices presenting the correlation among replicates of luminescence measurements for NanoLuc and NanoBiT libraries. The plots in the lower triangle of each matrix depict individual luminescence measurements for each replicate against the other replicates. Linear regressions are shown in gray.

The plots in the diagonal of each matrix depict data densities for each replicate. The heatmap in the upper triangle of each matrix depicts color-coded Pearson correlation coefficients (*R*).

B) Correlation matrix showing the relationship between prey protein abundance (measured by NanoLuc) and NanoBiT (NB) signals from Upf1, Cdc53, Met30, or Irc20. Correlations were calculated using the mean signal intensities from three experimental replicates.

C) Scatter plots showing NanoBiT signals from Upf1, Cdc53, Met30, and Irc20 baits plotted against prey protein abundance, which is estimated from the luminescence of the corresponding NanoLuc strains. Data are from three experimental replicates. The number of literature-curated experimental evidence for each protein pair is represented by colors (three or more, red; two, orange; one, yellow; zero, gray). Data points used for calculating classification metrics (Figures 2 and S3) are indicated in red and dark gray. Horizontal dashed lines indicate the highest NanoBiT value observed for putative non-interacting pairs.

D) Scatter plots showing NanoBiT ratios calculated with a single control bait (left) or multiple control baits (right). For the single control bait plots, the best correlated control bait is shown. For the multiple control baits plots, Met30 and Cdc53 were excluded as controls for calculating each other's ratios due to their known interaction in SCF^{Met30} complexes. Data are from three experimental replicates.

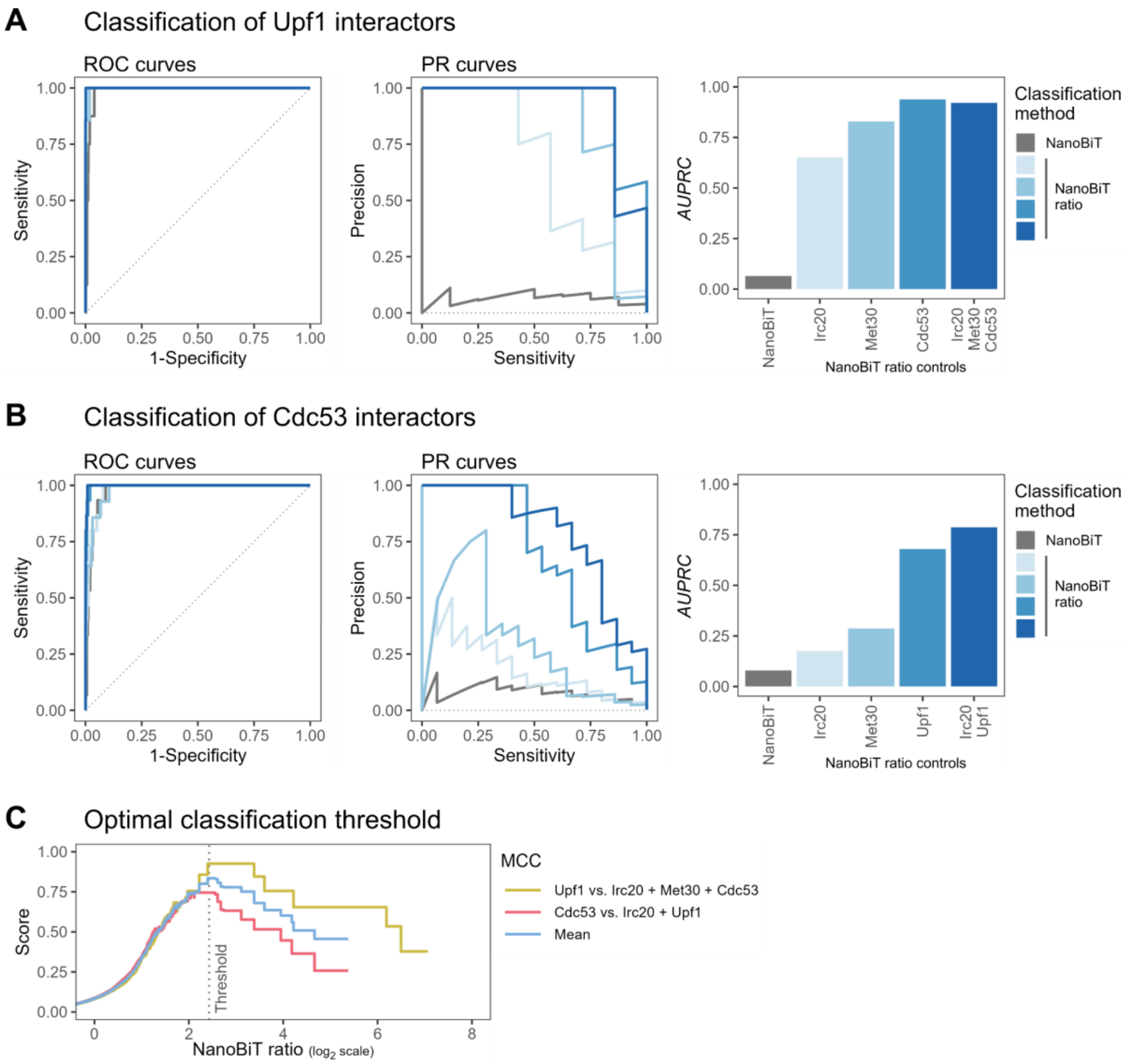

Figure S3. Classification of Upf1 and Cdc53 interactors, related to Figure 2.

A, B) Performance comparisons of Upf1's (A) and Cdc53's (B) interactor classifications. Receiver operating characteristic (ROC) and precision-recall (PR) curves were generated using NanoBiT signals (gray) or NanoBiT ratios (blues). The bar plots on the right display the area under the PR curve (*AUPRC*) for each classification. NanoBiT ratios were computed using the indicated baits as controls.

C) Step chart showing the Matthews correlation coefficient (MCC) at varying NanoBiT ratios for the best classifications of Upf1 and Cdc53 interactors. An optimal classification threshold was selected to correspond to the maximum MCC for both Upf1 and Cdc53.

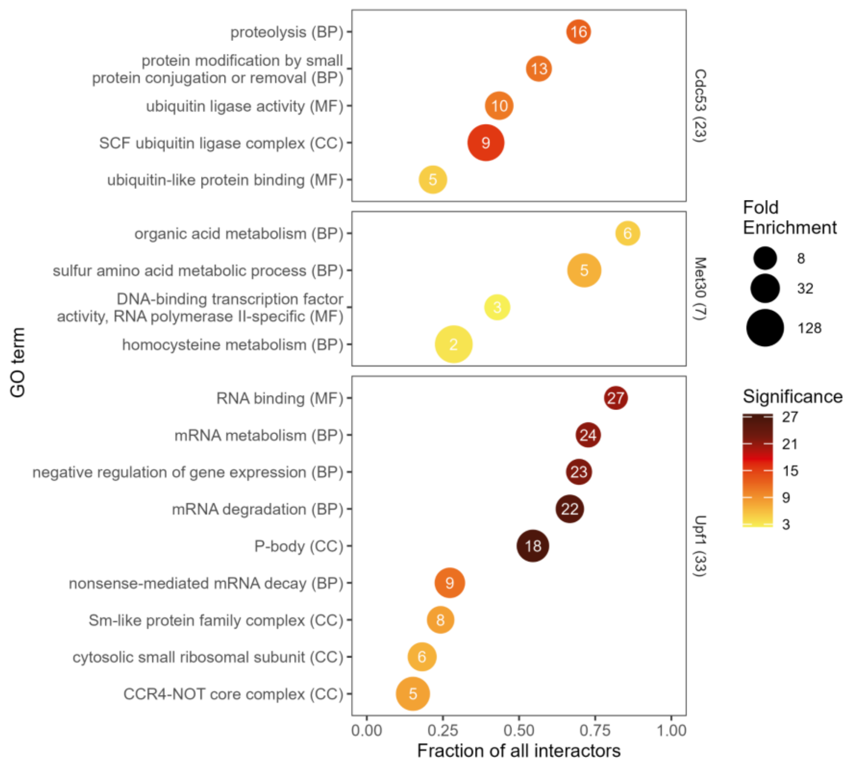

Figure S4. Gene ontology enrichment analysis, related to Figures 3 and 4.

Selection of GO terms significantly enriched among the identified interactors of Cdc53 (top), Met30 (middle) and Upf1 (down). The position of the dots along the x-axis represents the fraction of interactors annotated with the corresponding term (with the number of interactors shown in white within each dot). The size of the dot represents the fold enrichment, while the color indicates the significance of the enrichment (-log10(adjusted p-value)).

Table S1: Procedure and media used for the construction of the genome-wide strain libraries, related to STAR Methods

**Procedure**				
**Steps**	**Condition**	**Library**		
		**LgBiT-His and NanoLuc**	**SmBiT**	**NanoBiT**
Mating	1 day, 30 °C	YPD	YPD	YPD
Diploid selection	1 day at 30 °C, done twice	SC(MSG)<br>-Ura<br>+G418	SC(MSG)<br>-Ura<br>+clonNAT	YPD<br>+clonNAT/HygB
Sporulation	5-7 days at 25 °C	SPO	SPO	SPO
Haploid selection, step 1	2 days at 30 °C	SC(MSG)<br>-Ura/Leu/Arg/Lys<br>+Canavanine/Thialysine	SC(MSG)<br>-Ura/His<br>+5FC	SC(MSG)<br>-Leu/Arg/Lys<br>+Canavanine/Thialysine
Haploid selection, step 2	2 days at 30 °C, done twice	SC(MSG)<br>-Ura/Leu/ArgLys<br>+Canavanine/Thialysine<br>+HygB	SC(MSG)<br>-Ura/His/Lys<br>+5FC<br>+clonNAT	SC(MSG)<br>-Leu/Arg/Lys<br>+Canavanine/Thialysine<br>+clonNAT/HygB
Acceptor-donor module swapping	2 days at 30 °C, done twice	SC(MSG) Gal/Raf<br>-Leu	SC(MSG) Gal/Raf<br>-His	Not applicable
Acceptor module counterselection	2 days at 30 °C	SC(AS)+5FOA<br>-Leu	SC(AS)+5FOA<br>-His	Not applicable

**Media**	
**Name**	**Recipe**
SC(AS)+5FOA	1.7 g/L yeast nitrogen base without amino acids and ammonium sulfate, 5 g/L ammonium sulfate (AS), 2 g/L amino acid mix, 50 mg/mL uracil, 1g/L 5-Fluoroorotic acid (5FOA), 20 g/L glucose, 10 g/L agarose
SC(MSG)	1.7 g/L yeast nitrogen base without amino acids and ammonium sulfate, 1 g/L monosodium glutamic acid (MSG), 2 g/L amino acid mix, 20 g/L glucose, 10 g/L agarose
SC(MSG) Gal/Raf	1.7 g/L yeast nitrogen base without amino acids and ammonium sulfate, 1 g/L monosodium glutamic acid (MSG), 2 g/L amino acid mix, 20 g/L galactose, 20 g/L raffinose, 20 g/L agar
SPO	20 g/L potassium acetate, 20 g/L agar
YPD	10 g/L yeast extract, 20 g/L peptone, 20 g/L glucose, 10 g/L agarose

**Compounds for marker selection**	
**Name**	**Concentration for agarose plates**
Canavanine	12.5 mg/L
5-Fluorocytosine (5FC)	1.3 g/L

G418	100 mg/L
Hygromycin B (HygB)	75 mg/L
Nourseothricin (clonNAT)	25 mg/L
Thialysine	25 mg/L

References

[S1] Ho, Y., Gruhler, A., Heilbut, A., Bader, G.D., Moore, L., Adams, S.-L., Millar, A., Taylor, P., Bennett, K., Boutilier, K., et al. (2002). Systematic identification of protein complexes in *Saccharomyces cerevisiae* by mass spectrometry. Nature *415*, 180–183. https://doi.org/10.1038/415180a.

[S2] Gavin, A.-C., Aloy, P., Grandi, P., Krause, R., Boesche, M., Marzioch, M., Rau, C., Jensen, L.J., Bastuck, S., Dümpelfeld, B., et al. (2006). Proteome survey reveals modularity of the yeast cell machinery. Nature *440*, 631–636. https://doi.org/10.1038/nature04532.

[S3] Krogan, N.J., Cagney, G., Yu, H., Zhong, G., Guo, X., Ignatchenko, A., Li, J., Pu, S., Datta, N., Tikuisis, A.P., et al. (2006). Global landscape of protein complexes in the yeast *Saccharomyces cerevisiae*. Nature *440*, 637–643. https://doi.org/10.1038/nature04670.

[S4] Tarassov, K., Messier, V., Landry, C.R., Radinovic, S., Serna Molina, M.M., Shames, I., Malitskaya, Y., Vogel, J., Bussey, H., and Michnick, S.W. (2008). An in vivo map of the yeast protein interactome. Science *320*, 1465–1470. H https://doi.org/10.1126/science.1153878.

[S5] Michaelis, A.C., Brunner, A.-D., Zwiebel, M., Meier, F., Strauss, M.T., Bludau, I., and Mann, M. (2023). The social and structural architecture of the yeast protein interactome. Nature *624*, 192–200. https://doi.org/10.1038/s41586-023-06739-5.
